# Strategies for implementing long‐acting cabotegravir for PrEP in US clinics serving Black women: interim healthcare provider findings from the EBONI study

**DOI:** 10.1002/jia2.26497

**Published:** 2025-07-02

**Authors:** Katherine L. Nelson, Tammeka Evans Cooper, Yolanda Lawson, Dylan Baker, Satish Mocherla, Megan Dieterich, Theo Hodge, Alftan Dyson, Denise Sutherland‐Philips, Heidi Swygard, Lisa Petty, Peter Jeffery, Kenneth Sutton, Courtney Peasant Bonner, Sara M. Andrews, Samantha Chang, Piotr Budnik, Kimberly Smith, Annemiek de Ruiter, Maggie Czarnogorski, Nanlesta Pilgrim

**Affiliations:** ^1^ ViiV Healthcare Durham North Carolina USA; ^2^ MadeWell OBGYN Dallas Texas USA; ^3^ Emory University School of Medicine Atlanta Georgia USA; ^4^ Legacy Community Health Services Houston Texas USA; ^5^ Whitman‐Walker Institute Washington DC USA; ^6^ Washington Health Institute Washington DC USA; ^7^ GSK London UK; ^8^ RTI International Research Triangle Park North Carolina USA; ^9^ ViiV Healthcare London UK

**Keywords:** Black women, HIV prevention, implementation, long‐acting cabotegravir, pre‐exposure prophylaxis, sexual health

## Abstract

**Introduction:**

Long‐acting cabotegravir (CAB LA) is the first LA agent approved for HIV pre‐exposure prophylaxis. EBONI (NCT05514509) is a Phase 4 implementation study evaluating the implementation of CAB LA delivery to Black cis‐ and transgender (cis‐and‐trans) women in clinics located in the United States, including infectious disease (ID), primary care (PC) and women's health (WH) clinics. We present interim perspectives, considerations and strategies from healthcare professionals’ (HCPs’) experiences during the initial implementation stages of administering CAB LA.

**Methods:**

From August 2022 to June 2024, through quantitative surveys (prior to implementation [baseline] and Month 4 [M4]) and/or structured qualitative interviews (M4), HCPs provided their perceptions and experiences of integrating CAB LA in their clinical settings that served Black cis‐and‐trans women. Monthly implementation monitoring (IM) calls were also conducted. Survey data were analysed using descriptive statistics. Qualitative and IM data were coded and analysed using a Framework Analysis approach grounded in the Consolidated Framework for Implementation Research.

**Results:**

Ninety‐two HCPs across 20 sites completed baseline and M4 surveys; 57% were cisgender female and 43% were Black. HCPs across clinic types developed innovative approaches to support CAB LA implementation, with few HCPs (< 10%) reporting concerns about practice preparation. Initial HCP considerations related to patient adherence, insurance verification and patient identification reduced by M4 (absolute % reduction: 5–14%; 5–9%; and 4–12%, respectively). HCPs across clinic types serving Black women reported successful implementation strategies, including addressing medical mistrust and patient miseducation, staff training and reminder or tracking systems. Useful implementation strategies unique to clinic types included using electronic medical records to document whether patients were offered CAB LA (PC), designating specific days for administering injections (WH) and creating time for discussion with patients (ID).

**Conclusions:**

A range of strategies across clinics that routinely serve Black cis‐and‐trans women were used to support CAB LA implementation. Implementing CAB LA in clinical settings can be bolstered by addressing population‐specific concerns, increasing staff/patient education about CAB LA and modifying clinical flows. Lessons learned in EBONI can help support future integration for Black cis‐ and transgender women and provide valuable insights for various clinical environments.

**ClinicalTrials.gov number:**

NCT05514509

## 1 INTRODUCTION

Black cis‐ and transgender women are disproportionately affected by HIV in the United States. Black women accounted for 55% of new HIV diagnoses among cisgender women in 2019 but represent only 13% of the population [1]. Black transgender women represented 41% of new acquisitions among transgender women in 2022 [2]. Pre‐exposure prophylaxis (PrEP) coverage is significantly lower for Black people compared with White people in the United States (3.5 vs. 25.9 PrEP‐to‐need ratio) and for women compared with men (4.3 vs. 11.4 PrEP‐to‐need ratio) [3]. Only 15% of women who could benefit from PrEP have been prescribed PrEP compared with 40% in men [4]. The uptake of PrEP among Black cis‐ and transgender women has suffered for multiple reasons, including inadequate public health efforts to raise awareness about PrEP, low perceived likelihood of HIV acquisition, concerns about daily adherence and side effects, stigma, medical mistrust and a lack of specific marketing to Black cis‐ and transgender women [5, 6, 7]. Transgender women face additional barriers, including transphobia, lack of trans‐specific services/organizations and provider knowledge gaps [8, 9]. These issues are worsened by the failure to integrate PrEP services into community‐based and primary health systems where Black women seek care [10].

Long‐acting cabotegravir (CAB LA), administered every 2 months (Q2M) via intramuscular injection, is the first approved LA agent indicated for PrEP [11], representing a new paradigm for HIV prevention compared with daily oral PrEP. CAB LA has shown superiority in preventing HIV acquisition in pivotal trials (HPTN 083 and HPTN 084) and various real‐world studies [12, 13, 14, 15, 16, 17, 18, 19]. CAB LA is highly effective (HIV incidence 0.20 per 100 person‐years) and well tolerated in Black African women, as well as pregnant women [16, 19], with a small number of confirmed pregnancies and no congenital birth anomalies [19, 20].

CAB LA has several unique features that have the potential to increase PrEP uptake and reduce HIV incidence among Black cis‐ and transgender women. These include less frequent dosing, fewer adherence concerns, increased discretion, reduced stigma associated with PrEP [21], limited drug–drug interactions to facilitate use in broad populations and regular clinic visits Q2M for regular HIV RNA and sexually transmitted infection (STI) testing.

To help ensure that these benefits of CAB LA can be leveraged to reduce HIV health disparities among Black women, it is critical to integrate CAB LA into settings where they routinely seek care. This requires the integration and expansion of both traditional (e.g. infectious disease [ID] and primary care [PC] clinics) and newer (e.g. women's health [WH] clinics) points of care for PrEP [22]. Integrating the first injectable HIV prevention agent into clinical sites where PrEP has not been traditionally implemented, requires identifying and implementing strategies to increase awareness, incorporation into clinic workflows and working with insurance providers to increase access [23]. Identifying effective strategies for implementing CAB LA in different clinical settings may lead to increased use by Black cis‐ and transgender women, ultimately leading to a reduction in HIV acquisition rates among this demographic.

EBONI (NCT05514509) is a 12‐month Phase 4 implementation–effectiveness hybrid study evaluating the implementation and delivery of CAB LA across ID, PC and WH clinics across the United States that serve a large proportion of Black cis‐ and transgender women. Putting Black women's and community input into action, EBONI is the first industry‐led HIV implementation science study to take a gender‐aligned approach to recruitment via its inclusion of participants based on female gender identification versus sex assigned at birth. We present interim perspectives, considerations and strategies from healthcare professionals (HCPs) across different clinical settings (ID, PC and WH clinics) during the pre‐implementation and early implementation stages of integrating CAB LA for Black cis‐ and transgender women.

## 2 METHODS

### 2.1 Study design

**Figure 1:**
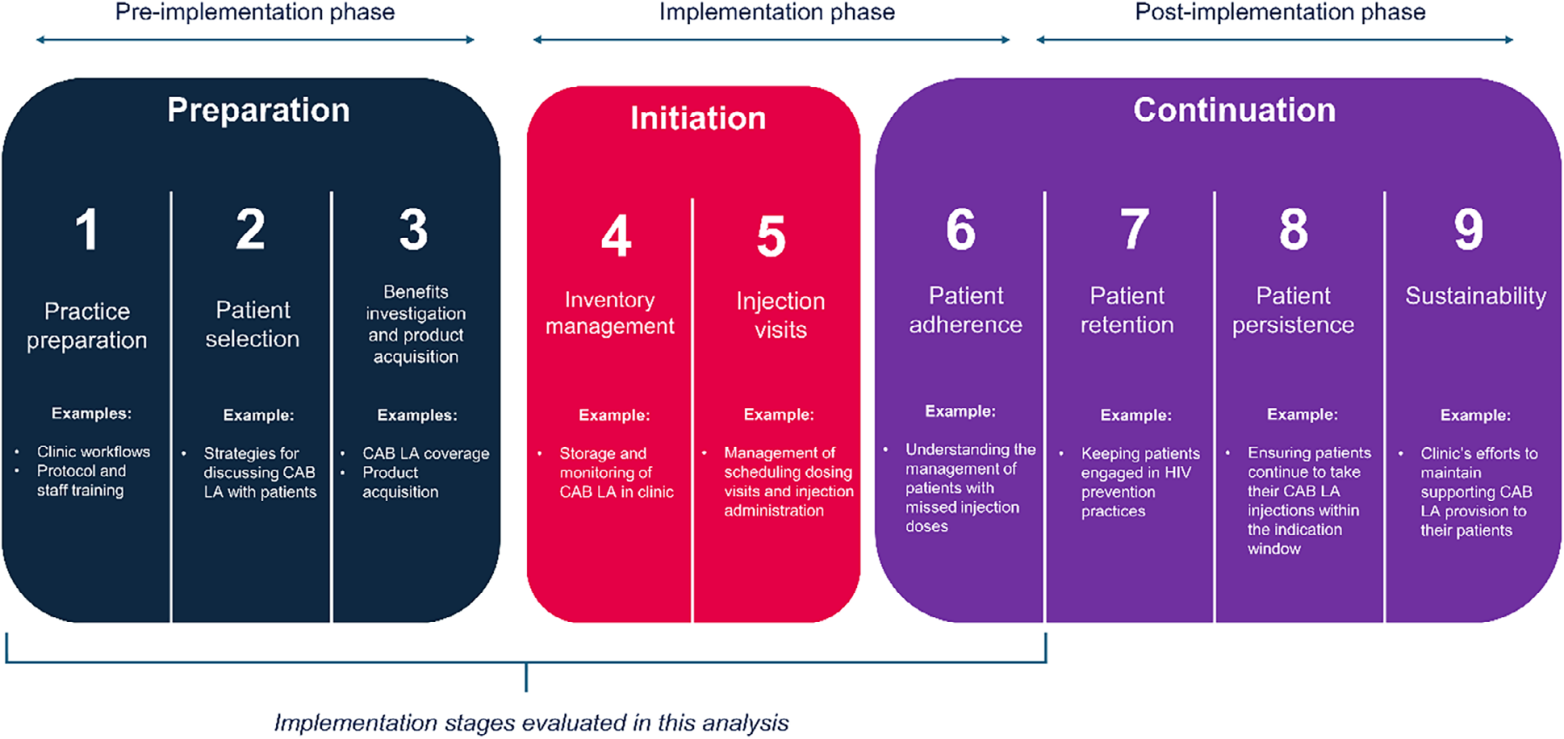
**Implementation stages of CAB LA integration**. Abbreviation: CAB LA, long‐acting cabotegravir.

Twenty clinics located in Ending the HIV Epidemic (EHE) jurisdictions [24] were identified and selected based on their Black cis‐ and transgender women patient population and contributed data to this interim analysis. Feasibility assessments of potential sites included clinic attestation that the demographic characteristics of a representative portion of their patient population were aligned to the study's target patient population. Clinic selection ensured different practice settings and provider types were included. This paper reports interim (up to and including Month 4/5; hereafter referred to as Month 4) perspectives, considerations and strategies from study staff participants (hereafter referred to as HCPs) across the different clinical settings collected during the pre‐implementation and early implementation phases of CAB LA (Figure 1).

Early and purposeful engagement with community members informed EBONI's study design and materials. The engagement included two advisory committees: one comprising WH providers and one with Black women leaders in organizations representing Black women [25]. The EBONI study protocol was subsequently reviewed and approved by a national, regional or investigational centre ethics committee or institutional review board.

### 2.2 Eligibility and enrolment

Eligible HCPs were employees responsible for administrative or clinical aspects of implementing or administering CAB LA. They held relevant medical licenses according to their role, were delegated the appropriate responsibilities by the site investigator and were able to understand and comply with protocol requirements, instructions and restrictions. Except for training on the protocol (e.g. assessments) and the implementation support available, no specific training was given to participating HCPs as part of EBONI.

#### 2.2.1 Outcomes and procedures

Endpoints assessed at Month 4 were HCPs’ barriers, facilitators, concerns and perceptions of CAB LA implementation, using a mixed methods approach. Quantitative outcomes were assessed via questionnaires and qualitative outcomes were assessed via interviews and monthly implementation monitoring (IM) calls. The sampling goal for quantitative questionnaires was approximately three to five HCPs per site, while the sampling goal for qualitative interviews was up to three HCPs per site.

#### 2.2.2 Quantitative outcomes

Quantitative data were collected at baseline and Month 4 (Figure S1), with HCPs completing electronic questionnaires on demographic characteristics, implementation outcomes and concerns or considerations relevant to CAB LA integration in their clinic. Concerns were assessed by asking HCPs to “…indicate how concerned [they] would be about each of the following being a barrier or challenge to delivering [CAB LA] to patients based on [their] current expectations and knowledge” using a 5‐point Likert scale (1 = extremely concerned, 5 = not at all concerned). In this analysis, the proportion of HCPs concerned with a potential challenge or barrier represents the proportion reporting “extremely/moderately concerned”; only challenges or barriers reported by > 10% of HCPs at baseline are reported as initial concerns in the text. For data on benefits verification and product acquisition, agreement with each statement was summarized as those who agreed (“completely agree/agree”), were indifferent (“neither agree nor disagree”) or disagreed (“completely disagree/disagree”).

#### 2.2.3 Qualitative outcomes

Qualitative data were obtained through semi‐structured interviews (∼60 minutes) conducted via video conferencing. Trained qualitative researchers conducted interviews approximately 4 months after the first participant, at each clinic, received their injection if no oral lead‐in was used, or approximately 5 months after the first study participant received their injection if an oral lead‐in was used. Interview topics were guided by the Consolidated Framework for Implementation Research (CFIR) [26] and Proctor implementation outcomes taxonomy [27], aiming to identify barriers and facilitators to CAB LA implementation, focusing on HCPs’ perceptions of PrEP implementation within their clinics. These included: feasibility of implementing CAB LA, implementation facilitators and considerations, helpful tools or strategies in CAB LA implementation and experiences with identifying suitable candidates for CAB LA and patient adherence. Interview questions were specifically focused on Black cis‐ and transgender women; however, discussion topics may have also been applicable to other populations.

In addition to interviewers, trained qualitative researchers facilitated monthly IM calls (∼30 minutes) with one to two HCPs per clinic, focused on implementation progress, barriers and solutions to implementation challenges. HCPs were asked what stage(s) of implementation (Figure 1) the clinic was focused on that month (adapted from Chamberlain et al.), and, if any concerns arose, what tools or strategies were used to address them [28]. Strategies were categorized based on whether they were unique to a specific clinic type or common across multiple clinic types.

### 2.3 Data analysis

#### 2.3.1 Quantitative analysis

The sample size was based on practical considerations in terms of the feasibility of enrolling an adequate number of sites, as well as HCPs and participants. Except for questions relating to benefits/product acquisition asked only at Month 4 (cross‐sectional sample), the analysis was conducted on a longitudinal sample comprising all HCPs who completed both baseline and Month 4 questionnaires. Descriptive summaries (means, medians, standard deviations, etc. for numeric data and proportions and counts for categorical data) were used to summarize and report quantitative data.

The same HCPs were expected to complete questionnaires at both time points and if an HCP left the study, the site was asked to replace them; however, replacement HCPs were not included in the longitudinal sample used to measure change in perception of CAB LA integration over time.

#### 2.3.2 Qualitative analysis

The 60‐minute semi‐structured qualitative interviews were recorded, transcribed and analysed in Nvivo12, a qualitative data analysis software, using a Framework Analysis Approach grounded in the CFIR [26, 29]. Content analysis was conducted following the steps described by Roller and Lavrakas [30]. A codebook was developed using constructs from CFIR and Proctor outcomes. After being trained on the codebook, three researchers coded seven transcripts in pairs. Coders met to discuss discrepancies and refine code definitions. After a kappa of ≥ 0.8 was demonstrated in the double‐coding of 10% of transcripts, the remaining transcripts were divided up to be coded by a single coder [31]. In the second phase of analysis, following the steps described by Roller and Lavrakas, themes were identified within categories (e.g. inner setting) and across subgroups of interest (e.g. clinic type and study arm). This allowed the data to be summarized and compared across participants, identifying overall themes and themes by subgroup.

Monthly monitoring calls were recorded and transcribed. Data from the transcription of the monthly IM calls were entered into a standard form in REDCap (a data‐reporting system) and synthesized by stage of implementation from across clinic sites. A summary report (*n* = 17) was created for each month then uploaded to and coded using NVivo12. A senior researcher developed a codebook with coding category definitions to capture themes including the stages of implementation, clinic characteristics and study tools or strategies. Two coders were trained on the codebook, provided input and applied the codebook independently to two monitoring reports. The coders met with the senior researcher to discuss each monitoring report, reach consensus on coding categories and achieve inter‐rater reliability (Cohen's kappa > 0.70). The remaining reports were divided between coders. Regular meetings were held to ensure alignment on coding and to discuss revisions to the codebook.

## 3 RESULTS

### 3.1 Clinic/HCP demographic characteristics

Overall, the 20 clinics participating in the study comprised federally qualified health centres (*n* = 3), hospitals (*n* = 3), non‐profit and community‐based organizations (*n* = 5), private clinics (*n* = 5), academic and research institutions (*n* = 2) and community health centres (*n* = 2). Clinic‐level characteristics are detailed in Table S1. Most were ID (ID or PC physician with a focus on HIV; *n* = 11), PC (*n* = 6) or WH clinics (*n* = 3). From these sites, 92 HCPs completed both baseline and Month 4 surveys (longitudinal sample). Overall, 57% (52/92) of HCPs were cisgender female and 43% (40/92) were Black (Table 1); the median (interquartile range) age was 44 years (35, 53). A total of 99 HCPs completed surveys at Month 4 (cross‐sectional sample; Table S2). A total of 72 HCPs also participated in qualitative interviews at Month 4.

**Table 1:** HCP demographics and characteristics (longitudinal and qualitative interview sample)

	Longitudinal sample (*n* = 92)	Qualitative interview sample (*n* = 72)
Gender identity, *n* (%)		
Cisgender male	26 (28.3)	23 (31.9)
Cisgender female	52 (56.5)	41 (56.9)
Transgender woman	0	1 (1.4)
Nonbinary	1 (1.1)	1 (1.4)
Other	6 (6.5)	2 (2.8)
Prefer not to answer	7 (7.6)	4 (5.6)
Age, median (interquartile range)	44.0 (35.0, 53.0)	43.5 (33.0, 52.0)
Race, *n* (%)		
Asian	3 (3.3)	3 (4.2)
Black	40 (43.5)	34 (47.2)
Mixed race	8 (8.7)	5 (6.9)
White	29 (31.5)	22 (30.6)
Native American	1 (1.1)	1 (1.4)
Other	5 (5.4)	4 (5.6)
Prefer not to answer	6 (6.5)	3 (4.2)
Role type, *n* (%)		
Physician	18 (19.6)	15 (20.8)
Advanced practice provider	17 (18.5)	13 (18.1)
Medical assistant	15 (16.3)	9 (12.5)
Administrator (office/clinic)	7 (7.6)	6 (8.3)
Nurse	10 (10.9)	9 (12.5)
Other role	25 (27.2)^a^	20 (27.8)^b^
Administers any type of injection, *n* (%)		
Yes	36 (39.1)	28 (38.9)
No	56 (60.9)	44 (61.1)
Prescribes medication, *n* (%)		
Yes	35 (38.0)	28 (38.9)
No	57 (62.0)	44 (61.1)
Currently provides PrEP counselling, *n* (%)		
Yes	60 (65.2)	49 (68.1)
No	32 (34.8)	23 (31.9)
Clinic provides CAB LA, *n* (%)		
Yes	73 (79.3)	59 (81.9)
No	9 (9.8)	8 (11.1)
I don't know	10 (10.9)	5 (6.9)
Medical specialty,^c^ *n* (%)		
HIV/infectious disease specialist	24 (70.6)	22 (81.4)
Internal medicine/primary care/general doctor/family practitioner	16 (47.1)	13 (48.1)
Women's health/OBGYN	2 (5.9)	3 (11.1)
Other^d^	2 (5.9)	1 (3.7)

Abbreviations: CAB, cabotegravir; HCP, healthcare professional; LA, long‐acting; OBGYN, obstetrics and gynaecology; PrEP, pre‐exposure prophylaxis.

^a^ Pharmacist (*n* = 3), social worker/case manager (*n* = 5), PrEP educator/navigator (*n* = 3) and other (*n* = 14).

^b^ Pharmacist (*n* = 1), social worker/case manager (*n* = 2), PrEP educator/navigator (*n* = 3) and other (*n* = 14).

^c^ Longitudinal sample: responses are among the *n* = 34 participants who responded “yes” to prescribing medications as part of their role. Qualitative interview sample: responses are among the *n* = 27 participants who responded “yes” to prescribing medications as part of their role. Participants could select ≥ 1 specialty.

^d^ Includes immunologists and other.

### 3.2 Perspectives, considerations and strategies of integrating CAB LA

**Figure 2:**
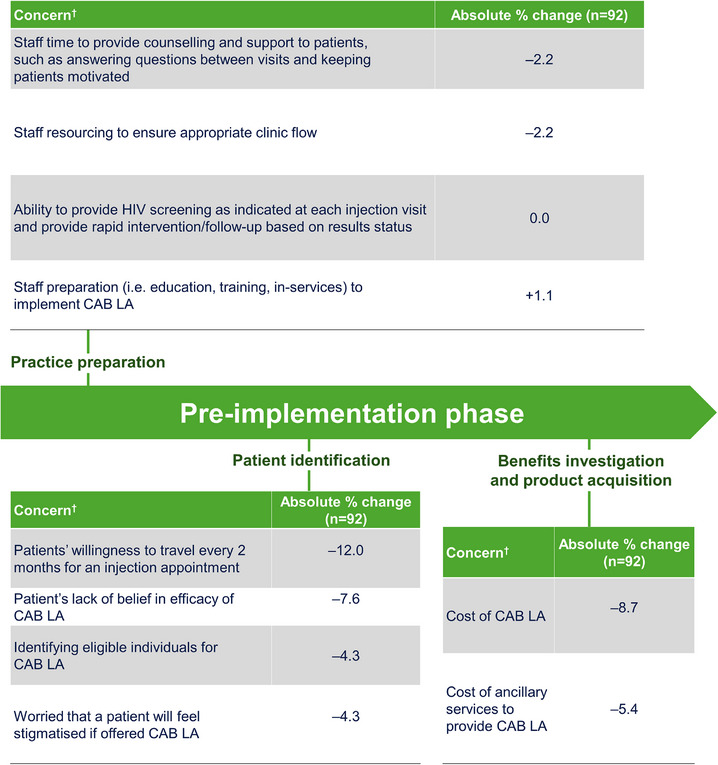
**HCP perceptions of pre‐implementation phase barriers before CAB LA implementation and 4 months into CAB LA implementation (longitudinal sample)**. Abbreviations: CAB, cabotegravir; HCP, healthcare professional; LA, long‐acting. ^†^Concerns were measured on a 5‐point Likert scale (1 = extremely concerned, 5 = not at all concerned). Results presented here were rated by HCPs as “extremely concerned” or “moderately concerned.” Absolute % change is the change in percentage points between baseline and Month 4.

**Figure 3:**
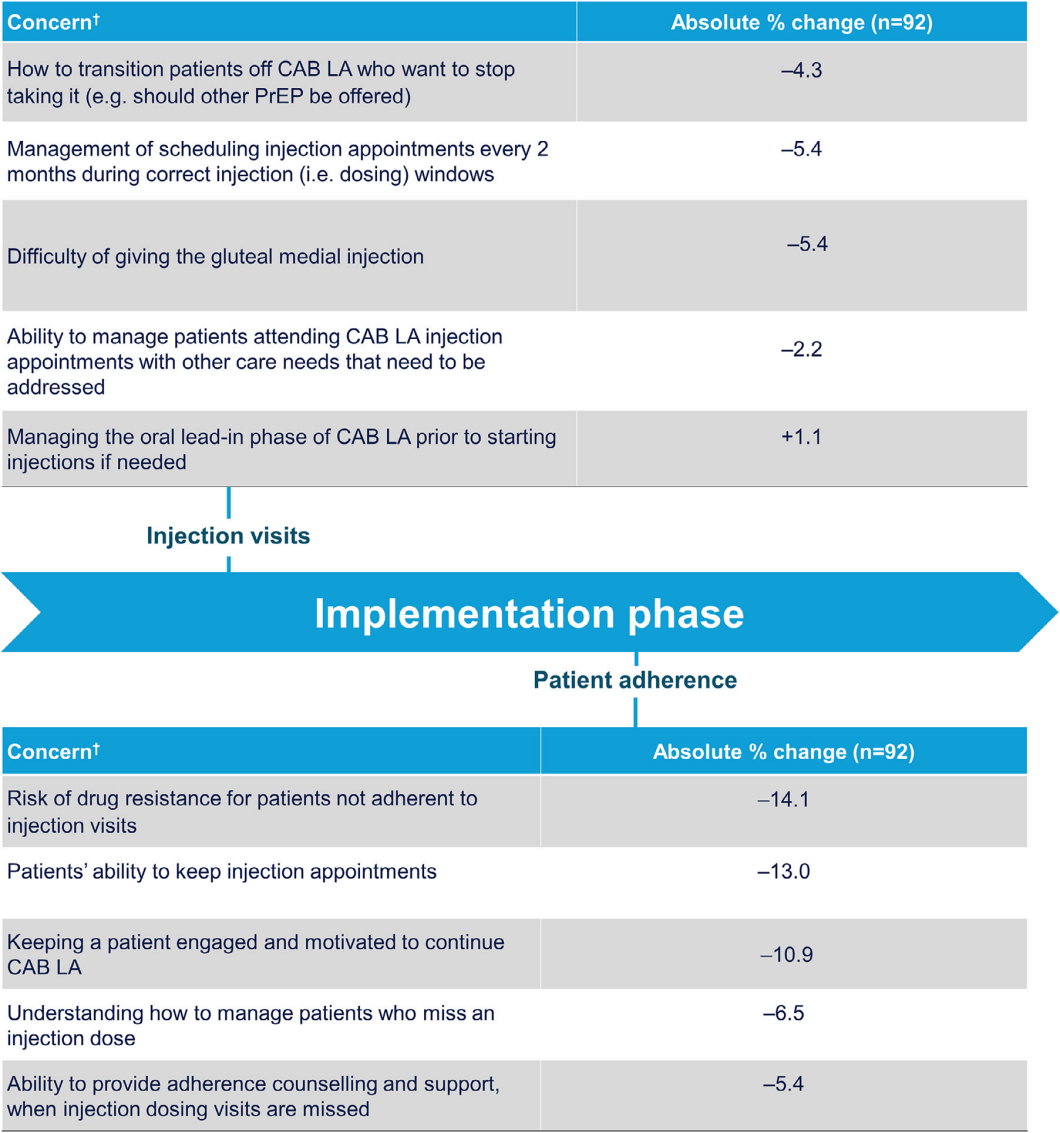
**HCP perceptions of implementation phase barriers before CAB LA implementation and 4 Months into CAB LA implementation (longitudinal sample)**. Abbreviations: CAB, cabotegravir; HCP, healthcare professional; LA, long‐acting; PrEP, pre‐exposure prophylaxis. ^†^Concerns were measured on a 5‐point Likert scale (1 = extremely concerned, 5 = not at all concerned). Results presented here were rated by HCPs as “extremely concerned” or “moderately concerned.” Absolute % change is the change in percentage points between baseline and Month 4.

At baseline, few HCPs (< 10%) reported concerns with the practice preparation stage of implementation (Figures 2 and 3 and Table S3). These included resourcing to ensure appropriate clinic flow and staff preparation. For the remaining stages of implementation, most initial implementation concerns decreased within the first 4 months of clinic integration of CAB LA. Although IM calls and interview data demonstrated that most considerations or strategies for CAB LA integration were consistent across different clinic types that served Black cis‐ and transgender women, several unique strategies for different clinic types were also identified (Figures S2 and S3). Top considerations and strategies to support the implementation of CAB are summarized in Table 2. Perspectives, considerations and strategies for CAB LA integration in clinics with a large client base of Black cis‐ and transgender women are summarized below by implementation stage.

**Table 2:** Top considerations and strategies to support the implementation of CAB LA

Strategy	Implementation stage	Number of clinics reporting the strategy
Designate a secured space for injection storage	Practise preparation	16
Build relationships with other clinics/community‐based organizations that may serve people who would benefit from PrEP	Patient identification	14
Utilize, redesignate or hire a benefits verification team/individual or medical billing team/individual at the clinic or health system	Benefits investigation and product acquisition	13
Train multiple staff on procedures^a^ and materials^b^ before the first patient receives CAB LA	Practise preparation	8
Leverage staff with prior health education and experience	Patient identification	5
Conduct regular records review to determine eligible patients	Patient identification	5
Send reminders and facilitate appointment scheduling through various channels^c^	Patient adherence	5
Determine where injections will be administered and tests will be taken/processed	Practise preparation	4

Abbreviations: CAB, cabotegravir; EMR, electronic medical record; LA, long‐acting; PrEP, pre‐exposure prophylaxis.

^a^ That is injections, testing.

^b^ That is EMR, workflow updates.

^c^ Text, email, etc.

#### 3.2.1 Pre‐implementation phase of CAB LA

##### 3.2.1.1 Practice preparation

Few HCPs (< 10%) reported having any concerns around individual aspects of practice preparation; this was maintained at Month 4. During implementation calls, HCPs highlighted staff training and education as important for preparing their clinics to deliver CAB LA and having a positive impact on CAB LA integration in their clinic that served Black cis‐ and transgender women. HCPs specifically mentioned training staff on procedures and materials prior to their first patient receiving CAB LA, training staff on different workflows and training multiple staff to administer CAB LA as a strategy to relieve workload:

“…I think our planning committee just made sure we were very fine‐tuned in every detail that we needed for CAB LA before we implemented it… That made things easier once we implemented it in the practice because we knew exactly what to do at that point, and everyone was trained on each workflow… So just having that workflow, having those protocols in place before we implemented it in the practice made it easier and smoother.” (Administrator, WH)

Some HCPs discussed developing new implementation processes or adjusting existing processes for CAB LA integration, such as defining standard operating procedures for test processing or redesignating staff to support during CAB LA integration. Several HCPs also discussed the importance of having adequate physical and personnel resourcing (e.g. space for injections, storage space and dedicated or adequate staff for CAB LA integration). HCPs also identified leadership designation of resources for CAB LA as important for integration:

“And our medical director and associate medical director are behind this 100 percent. I think it's just having the staff, the director, and pharmacy all in it to make it successful.” (Advanced practice provider, PC)

Pre‐determining insurance and reimbursement pathways was also highlighted as a strategy by multiple clinic types, specifically whether to use specialty pharmacies or buy‐and‐bill options (whereby providers order and pay for the medication and receive reimbursements from third‐party payers). Notably, WH clinics underlined the importance of selecting consistent days to give injections. One HCP at a WH clinic mentioned needing to add a new billing code and understand the new workflow associated with getting injections from the pharmacy.

“So there's a specific code that's used to bill for it. And before the patient comes in, we know which insurance. We develop a grid to figure out which insurance needs authorization or not… We have a process. We have protocols in place regarding anything in the practice. There are protocols in place to verify that before the patient comes in.” (Administrator, WH)

##### 3.2.1.2 Patient identification

The initial concerns expressed by HCPs at baseline around aspects of patient identification decreased by Month 4. These included patients’ willingness to travel Q2M for an injection appointment (–12.0%), patients’ lack of belief in CAB LA efficacy (–7.6%) and their own ability to identify eligible individuals for CAB LA (–4.3%). Fewer HCPs (7.6%) at baseline were concerned that a patient may feel stigmatized if offered CAB LA; however, this concern also decreased by Month 4 (–4.3%).

Qualitative data described HCPs’ two main perspectives used to identify Black cis‐ and transgender women for CAB LA: offering PrEP to all sexually active women; or using a shared decision‐making model and a specific list of criteria. For the latter, such criteria included considering insurance coverage, housing circumstance, their partner's behaviours and their medical history. For example, those who adhere to medical visits but have difficulty with daily pill‐taking were described as ideal candidates.

“…we try to prioritize patients who have poor adherence to oral PrEP, have side effects from oral PrEP, or have contraindications for oral PrEP. We do put a lot of emphasis on kind of making sure that patients that are starting on CAB LA are going to be able to make their appointments.” (Physician, PC)

Specifically, transgender women were identified as good candidates for CAB LA due to the reduced pill burden for those engaged in hormone therapy. Other HCPs drew comparisons between CAB LA and contraception, highlighting that patients who have utilized injectable contraceptive may be a facilitator for CAB LA uptake among women.

Common strategies used across multiple clinic types to mitigate patient identification barriers for Black cis‐ and transgender women included building relationships with other clinics or community‐based organizations [25], distributing electronic print materials for patient education on CAB LA, sexual health or both to build confidence in CAB LA efficacy and leveraging staff with prior experience with CAB LA.

When looking at considerations and strategies by different clinic types to address HCP challenges in patient identification, ID clinics reported working with onsite pharmacies to identify patients interested in alternative PrEP options and providing information about CAB LA to patients via the clinic/health system patient portal. PC clinics reported administering a questionnaire to patients on oral PrEP to gauge interest in CAB LA or trying to ascertain interest in CAB LA at new patients’ first appointments to mitigate the challenge of limited staff time for patient identification. WH clinics used roleplay in preparation for discussing CAB LA with patients as a strategy to address any misunderstandings of the treatment and medical mistrust. HCPs also discussed the importance of establishing an open, honest and comfortable patient–provider relationship before introducing CAB LA and framing CAB LA as a tool for empowerment for Black cis‐ and transgender women compared with focusing solely on reducing risk.

##### 3.2.1.3 Benefits investigation and product acquisition

Initial concerns regarding the cost of CAB LA and the cost of ancillary services to provide CAB LA expressed by HCPs at baseline decreased by Month 4 (–8.7% and –5.4%, respectively). Overall, 61.8% of HCPs completing the Month 4 survey (cross‐sectional dataset) reported that completing the benefits enrolment was easy, and 41.2% were satisfied with the benefits verification process (Table 3).

**Table 3:** HCP perceptions of benefits verification and product acquisition at Month 4 (cross‐sectional sample)

*n* (%)	Month 4 (*N* = 99)
Involvement in any part of the acquisition process for CAB LA	51 (51.5)
Methods of acquisition of CAB LA^a,b^	
Buy‐and‐bill^c^	21 (41.2)
White bagging^d^	34 (66.7)
Clear bagging^e^	17 (33.3)
Other	2 (3.9)
I don't know	7 (13.7)
On average, how long has it taken to receive confirmation of benefits verification for CAB LA?^a^	
1–2 days	6 (11.8)
3–4 days	8 (15.7)
5–6 days	5 (9.8)
7–8 days	6 (11.8)
9–10 days	5 (9.8)
11–15 days	7 (13.7)
16–20 days	3 (5.9)
20+ days	2 (3.9)
I don't know	9 (17.6)
On average, how long has it taken for patients to receive CAB LA from the point of benefits verification?^a^	
1–2 days	3 (5.9)
3–4 days	4 (7.8)
5–6 days	1 (2.0)
7–8 days	10 (19.6)
9–10 days	5 (9.8)
11–15 days	7 (13.7)
16–20 days	4 (7.8)
20+ days	10 (19.6)
I don't know	7 (13.7)
Completing the benefits enrolment forms is easy^f^	
Completely disagree/disagree	3 (8.8)
Neither agree nor disagree	10 (29.4)
Completely agree/agree	21 (61.8)
I am satisfied with the benefits verification process^f^	
Completely disagree/disagree	9 (26.5)
Neither agree nor disagree	11 (32.4)
Completely agree/agree	14 (41.2)
It has been easy to acquire CAB LA for patients^f^	
Completely disagree/disagree	14 (41.2)
Neither agree nor disagree	6 (17.6)
Completely agree/agree	14 (41.2)
I know what to do if I am having trouble acquiring CAB LA for patients^f^	
Completely disagree/disagree	4 (11.8)
Neither agree nor disagree	2 (5.9)
Completely agree/agree	28 (82.4)

Abbreviations: CAB, cabotegravir; HCP, healthcare provider; LA, long‐acting; PrEP, pre‐exposure prophylaxis.

^a^ Responses are among participants who reported being involved in any part of the acquisition process for CAB LA (*n* = 51).

^b^ Participants were allowed to select more than one response.

^c^ Buy‐and‐bill refers to when providers order and pay for the medication and receive reimbursements from third‐party payers.

^d^ White bagging refers to when a specialty pharmacy fills a prescription and sends the medication to an HCP for administration to a patient.

^e^ Clear bagging refers to when a healthcare system's internal specialty pharmacy dispenses and delivers medication to a healthcare provider for administration to a patient.

^f^ Responses are among participants who reported personally using, or the clinic using, patient support programmes (*n* = 34).

During the qualitative interviews, HCPs reported that the CAB LA acquisition process was largely driven by a patient's insurance carrier, specifically when choosing which pharmacy to fill the medication at. HCPs also discussed insurance challenges, including prior authorization denials without rationale and the impact delayed benefits verification could have on patients’ interest in CAB LA.

Some HCPs discussed difficulties in benefit reimbursement using the buy‐and‐bill option; however, other HCPs discussed that using buy‐and‐bill greatly facilitated the process of acquiring CAB LA quicker. Some HCPs indicated that they switched from other processes (e.g. specialty pharmacy) to buy‐and‐bill, with a more streamlined result.

“If we just have 1 or 2 streamlined pharmacies, we know they're going to take care of all the verification, they'll sort out all benefits. They can take both medical and pharmacy benefits. They send us the medication.” (Physician, PC)

Other clinics preferred to use a specialty pharmacy to help facilitate prior authorizations or billing and medication acquisition. However, insurances differed in the coverage of CAB LA using specialty or on‐site pharmacies. Clinics used different methods to address the CAB LA acquisition process, such as getting the medication shipped through a retail pharmacy or “drop shipped” from other specialty pharmacies.

Common strategies reported by multiple clinic types to help with benefits investigation included leveraging or designating a dedicated benefits verification or medical billing team or individual, verifying co‐pay assistance options and using a helpline/live support to navigate buy‐and‐bill or product acquisition. Different clinics also reported unique strategies to address considerations around the varying length of benefits verification processes and difficulties in benefit reimbursement. WH clinics called patients and scheduled injections before CAB LA was physically on site and contacted the vendor directly to discuss shipping delays. WH clinics also highlighted the importance of building relationships with specialty pharmacies/pharmacists. A PC clinic developed a justification letter template that could be modified by patients to help with the benefits verification process.

#### 3.2.2 Implementation phase of CAB LA

##### 3.2.2.1 Inventory management

Clinics discussed the potential for supply management issues to lead to injection delays and suggested using staff to oversee inventory management or designating specific days to order CAB LA for the upcoming month as supporting strategies. One PC clinic called mail‐order or independent pharmacies on behalf of patients to coordinate shipment of CAB LA directly to the clinic, while other PC clinics stocked a pre‐determined quantity of injections:

“… they had to do a protocol on how to organize a fridge, how we can set up a system where we know what medicines we have on stock, whose medicines we have on the stock… we had to… have a real constant line of communication in order to make sure that we have the medications arrived, they're in the fridge, they're ready to go, they're there not here, we need to get it.” (Other, HIV Specialist)

##### 3.2.2.2 Injection visits

HCPs described the step‐by‐step process of the injection visits, including how clinics conducted screening tests, administered the injection and observed the patient after the injection. Some HCPs reported additional processes, with one HCP at a WH clinic performing a full panel STI screening, including a rapid HIV and/or a PCR test to assess viral load, and administering the injection on the same day.

“As somebody who gives an injection myself, it's very straightforward [this] device that they give you in the packaging, that you just attached to put to draw up, it's very nice.” (Advanced practice provider, HIV Specialist)

At baseline, HCPs expressed initial concerns about knowing how to transition patients off CAB LA; however, this decreased (–4.3%) by Month 4. Specific considerations by clinic type included the time it takes to schedule patients and a lack of trained injection delivery staff limiting appointment availability for ID and WH clinics, respectively. To address such concerns, HCPs spoke about the advantages of having injection‐only visits and implementing flexible clinic hour. This helped to support providers by reducing their workload on regular clinic days, and it also helped to accommodate patients who must work during the day and could not get to their appointments until after normal business hours.

“Our office has a late day… we work actually today till 7… the patient may say, well, I can't come up there because I have to work late this day… And so, they're pretty good about letting us know. And then we have it sent here [from the pharmacy], and we're able to see some patients who can't make it till like 5:30 or 6 because we want to make sure that we don't miss their injection date.” (Other, HIV Specialist)

Other supporting strategies by specific clinic type included delineating staff responsibilities during injection visits (e.g. testing, injecting scheduling), establishing a direct line of communication for patients to communicate scheduling changes, creating time for discussion with patients and lengthening appointment time slots for ID clinics, training staff on how to administer HIV rapid tests, setting up the HIV testing machine prior to patient arrival and designating a specific injection day for WH clinics. PC clinics suggested using electronic medical records for laboratory tests to streamline ordering and building in additional time at the end of patient visits to monitor post‐injection effects as supporting integration strategies.

##### 3.2.2.3 Patient adherence

At baseline, HCPs expressed initial concerns around individual aspects of patient adherence that decreased by Month 4, including the risk of drug resistance for patients not adherent to injection visits (–14.1%), patients’ ability to keep injection appointments (–13.0%), keeping patients engaged and motivated to continue CAB LA (–10.9%) and understanding how to manage patients who miss an injection (–6.5%).

HCPs discussed the concern of their Black cis‐ and transgender women patients missing scheduled appointments, offering common mitigating strategies across clinic types. One HCP specifically mentioned the helpfulness of keeping walk‐in appointments as an option:

“I don't think that it has. One of the things that we pride ourselves on is trying to keep a certain amount of open appointments, for walk‐ins…” (Advanced practice provider, HIV Specialist)

HCPs also explained that they acted promptly and diligently to follow up with patients who miss their injection appointments to avoid having to restart the medication:

“That's why I like to give them all the information upfront in regard to how important it is for them to make it to their appointments. Because then at that point I'm like, ‘Well, if you can't make it, then why are we getting you on something that could possibly make you resistant to the HIV or get you a resistant virus?’” (Other, PC)

Considerations were also discussed by specific clinic types. For example, PC clinics discussed patients initiating care at new clinics and highlighted a supporting strategy of offering oral PrEP as an option for situations outside the clinic's control (e.g. insurance delays). ID clinics mentioned several supporting strategies; this includes considering that sending multiple reminders can overwhelm patients, and scheduling the next appointment prior to leaving the clinic and offering testing the same day as the injection appointment.

Related to the impact of side effects on adherence, HCPs also reported that side effects were minimal for their Black cis‐ and transgender women patients and mainly comprised injection site reactions, with very few patients discontinuing due to side effects. Some noted that the lack of gastrointestinal discomfort, which is associated with oral PrEP (tenofovir disoproxil fumarate [TDF]), may be appealing to patients; this was thought to be particularly important for cisgender women whose only previous PrEP option was TDF.

“Some of them mentioned that previously they'd been on the pill, and it caused some gastric problems. So they're hoping that with this medication, those will disappear.” (Other, HIV Specialist)

## 4 DISCUSSION

This ongoing Phase 4, mixed‐methods, implementation–effectiveness hybrid study is being conducted at ID, PC and WH clinics across the United States serving Black cis‐ and transgender women to evaluate the implementation of CAB LA delivery in these specific populations. As the first injectable HIV prevention modality, CAB LA requires changes to the conventional routine of prescribing oral PrEP therapy [32]. In EBONI, HCPs identified multiple considerations and replicable strategies to mitigate challenges at the pre‐implementation and implementation stages of integrating CAB LA into standard of care for Black cis‐ and transgender women. The considerations and strategies spanned workflow, staffing needs, scheduling, billing and training.

In EBONI, multiple clinic types serving Black cis‐ and transgender women reported considerations around patients’ medical mistrust, miseducation or lack of education, which is an observation supported by other studies reporting lower levels of PrEP knowledge among these populations [6, 33, 34]. Black cisgender women have also reported that providers with whom they have established a trusting relationship are the best sources of information about PrEP [35]. Therefore, the strategies suggested by HCPs participating in EBONI relating to patient identification, leveraging staff with prior health education experience, patient education materials and building relationships with other clinics/community‐based organizations, may be key to helping combat medical mistrust and increase the uptake of PrEP in this specific population.

In addition to strategies addressing medical mistrust and miseducation, other strategies were identified that are applicable for clinics serving Black cis‐ and transgender women, as well as a wide range of clinic types preparing to adopt CAB LA. For example, multiple clinic types mentioned operational considerations, including staff training, designating physical spaces for storage and injection delivery, changes to insurance and inventory management and setting up appointment reminders and scheduling processes. These findings align with previous implementation research on CAB LA integration [36, 37, 38]. It is also encouraging that various clinical sites implemented similar strategies, demonstrating their potential to support wider scale‐up efforts. Unique implementation considerations and strategies were also reported by clinic type, reflecting challenges and solutions arising from the distinct operating environments of ID, PC and WH clinics. These findings can improve the integration of CAB LA by offering context‐dependent implementation solutions that can be used by similar clinic types experiencing parallel challenges.

Prior research highlighted potential implementation concerns regarding CAB LA for PrEP and LA injectable treatment for HIV for underrepresented populations, many of which were also noted in this study for Black cis‐ and transgender women. These occurred at the drug (e.g. drug resistance, drug–drug interactions, frequency of dosing, associated costs and injection‐site reactions), patient (e.g. medical mistrust, stigma, fear of side effects and a lack of awareness), provider (e.g. patient selection) and system level (e.g. training, resourcing, procurement, insurance considerations and storage) [36, 39, 40, 41, 42]; however, few studies assessed how perceptions of these barriers changed throughout and/or at post‐implementation. EBONI demonstrated that common implementation concerns decreased within‐person by Month 4 for HCPs caring for Black cis‐ and transgender women as they gained more experience with CAB LA, supporting findings from broader populations in the CARISEL and CUSTOMIZE studies evaluating the implementation of CAB + rilpivirine LA for HIV treatment [43, 44].

In summary, clinics interested in implementing CAB LA can consider incorporating some of the strategies reported in this study to optimize their own implementation journey. Key strategies that could be used are summarized in Table 2.

This analysis has some limitations that should be acknowledged. During qualitative interviews, not all probes were asked of all HCPs (e.g. due to a lack of time). Additionally, owing to the nature of the data capture, HCPs did not necessarily discuss each theme and instead discussed the themes most relevant to their experience. Furthermore, as the study was conducted exclusively in the EHE region of the United States, some of the considerations and strategies may not be relevant to clinics in other countries, such as the data reported around product acquisition and benefits verification, which are region‐specific. Additionally, as this was an interim analysis, data beyond the first 4 months of implementation of CAB LA are required to determine if additional implementation strategies were adopted, or initial strategies modified.

## 5 CONCLUSIONS

Clinics can implement multiple strategies to support the implementation of CAB LA in specific patient populations. HCPs’ implementation concerns decreased during the first 4 months of the pre‐implementation and early implementation stages of CAB LA administration to Black cis‐ and transgender women. Despite some considerations and strategies potentially being more relevant to Black cis‐ and transgender women, such as medical mistrust, miseducation and patient identification, many are widely applicable across the healthcare spectrum and broader populations, including clinical staff training, designating physical and personnel resources and appointment reminders. These results suggest that CAB LA can be implemented into different healthcare settings and support the equitable scale‐up for potential increased access to CAB LA in Black cis‐ and transgender women. Subsequent research should focus on the sustainability of the implementation strategies identified and transferability to other populations, as well as how they can be scaled across various healthcare settings.

## COMPETING INTERESTS

KLN, TEC, AD, DS‐P, HS, LP, KSu, PB, KSm, AdR, MC and NP are employees of ViiV Healthcare and stockholders in GSK. YL declares grant support from ViiV/GSK, payment/honoria for participation as an Endometrial Cancer Panellist, JAMA Precision Medication Education and Pfizer COVID‐19 Vaccine educational content, travel support from Gilead, advisory board participant for Gilead and ViiV Healthcare and Immediate Past President for the National Medical Association. DB reports grant support from ViiV Healthcare during the course of the present manuscript (paid to institution), support from ViiV Healthcare/GSK for travel to ID Week 2024 to present the EBONI study findings. SM serves as a board member of AAHIVM Texas Chapter. MD reports support from ViiV Healthcare/GSK for travel to the EBONI research summit. TH has nothing to disclose. PJ was an employee and stockholder of GSK during the conduct of the study. CPB, SMA and SC are employees of RTI, a vendor paid by GSK/ViiV Healthcare to conduct the EBONI study.

## AUTHORS’ CONTRIBUTIONS

All authors vouch for the accuracy and completeness of the data, data analyses and interpretation and fidelity to the protocol.

## FUNDING

This study was funded by ViiV Healthcare. The funders participated in the collection, analysis and interpretation of data; in the writing of the report; and in the decision to submit the paper for publication.

## Supporting information




**Figure S1**: Timing of assessments for HCPs
**Figure S2**: Considerations and strategies to support the implementation of CAB LA in the pre‐implementation phase
**Figure S3**: Considerations and strategies to support the implementation of CAB LA in the implementation phase
**Table S1**: Clinic‐level characteristics
**Table S2**: HCP demographics and characteristics (cross‐sectional sample)
**Table S3**: HCP perceptions of implementation barriers before CAB LA implementation and 4 Months into CAB LA implementation (longitudinal sample)

## Data Availability

Data‐sharing requests will be considered by the management group upon written request to the corresponding author. Deidentified participant data or other prespecified data may be available subject to a written proposal and a signed data‐sharing agreement.
