# Supplementary material for: Strategies for implementing long‐acting cabotegravir for PrEP in US clinics serving Black women: interim healthcare provider findings from the EBONI study
Source: J Int AIDS Soc. 2025 Jul 2;28(Suppl 2):e26497. doi: 10.1002/jia2.26497 (PMC12215822; doi:10.1002/jia2.26497)
Supplement: Supplementary file 1 — Figure S1: Timing of assessments for HCPs Figure S2: Considerations and strategies to support the implementation of CAB LA in the pre‐implementation phase Figure S3: Considerations and strategies to support the implementation of CAB LA in the implementation phase Table S1: Clinic‐level characteristics Table S2: HCP demographics and characteristics (cross‐sectional sample) Table S3: HCP perceptions of implementation barriers before CAB LA implementation and 4 Months into CAB LA implementation (longitudinal sample) [file JIA2-28-e26497-s001.docx]

# Supplement

## Supplementary Figure 1. Timing of assessments for HCPs


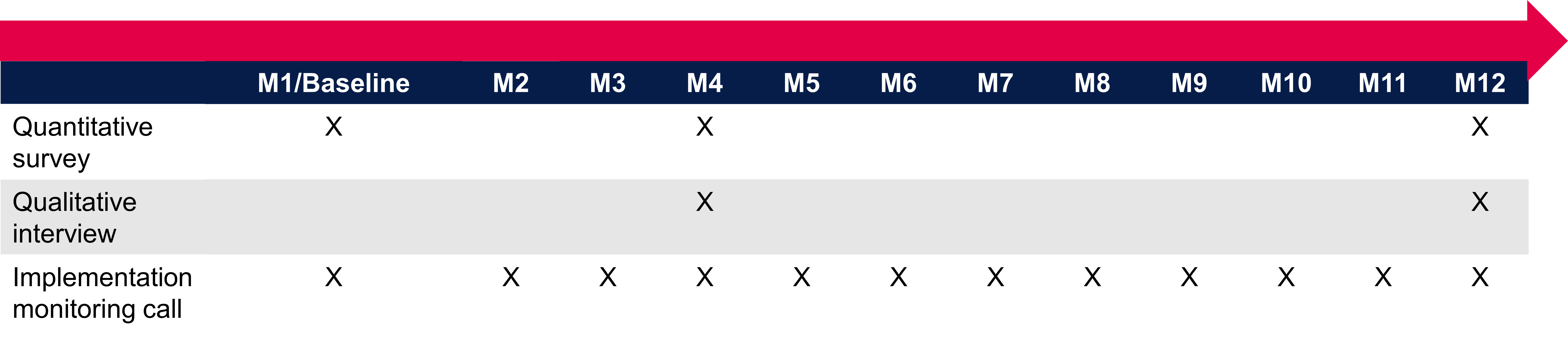


HCP, healthcare professional; M, Month.

## Supplementary Figure 2. Considerations and strategies to support the implementation of CAB LA in the pre-implementation phase

^
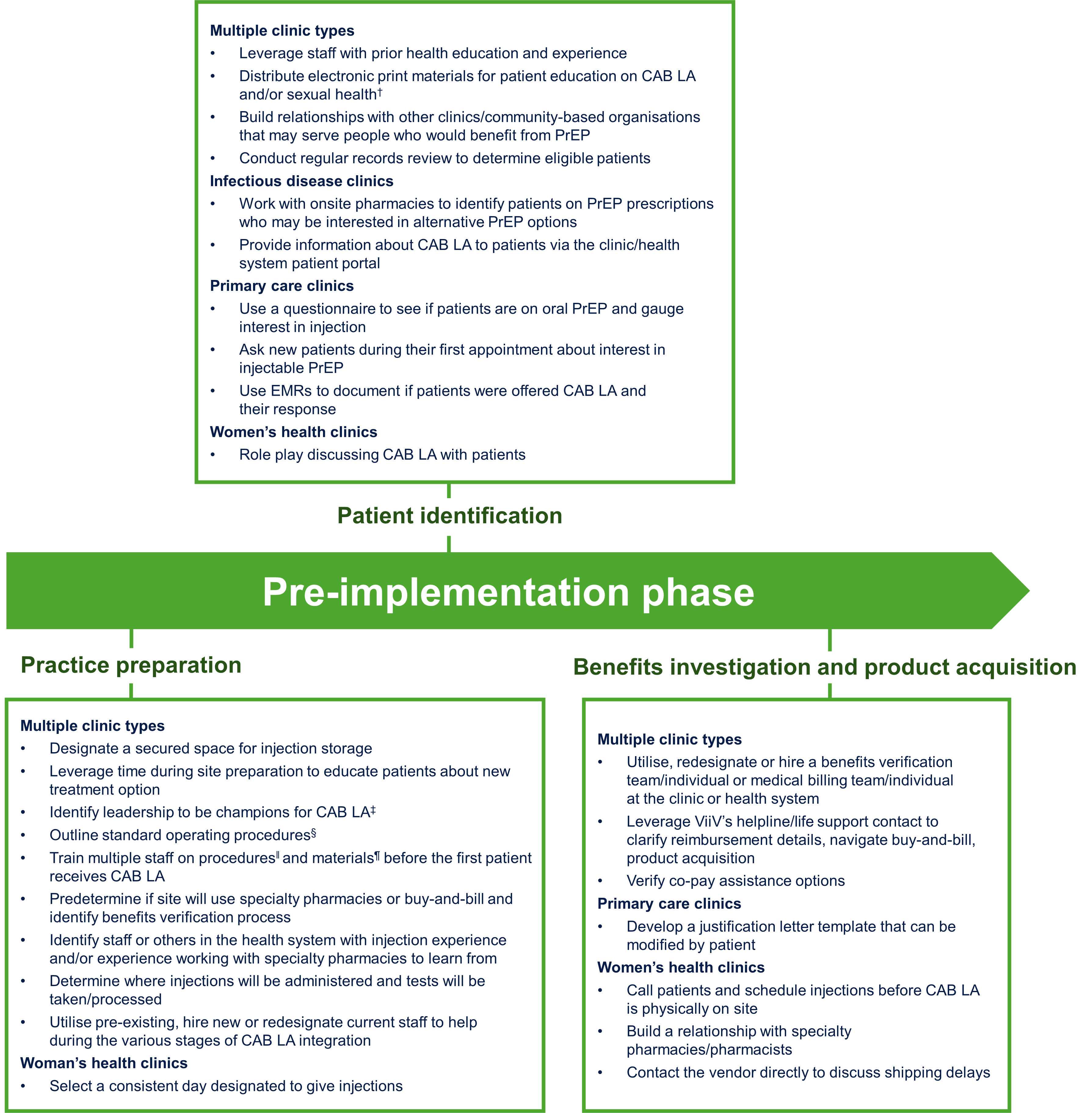
^

^†^I.e. newsletters, brochures, pamphlets, posters, flyers. ^‡^I.e. support use, designate resources. ^§^I.e. how to schedule /reschedule, visit content, medication acquisition. ^‖^I.e. injections, testing.
^¶^I.e. EMR, workflow updates.
CAB, cabotegravir; EMR, electronic medical record; LA, long-acting; PrEP, pre-exposure prophylaxis.

## Supplementary Figure 3. Considerations and strategies to support the implementation of CAB LA in the implementation phase

**
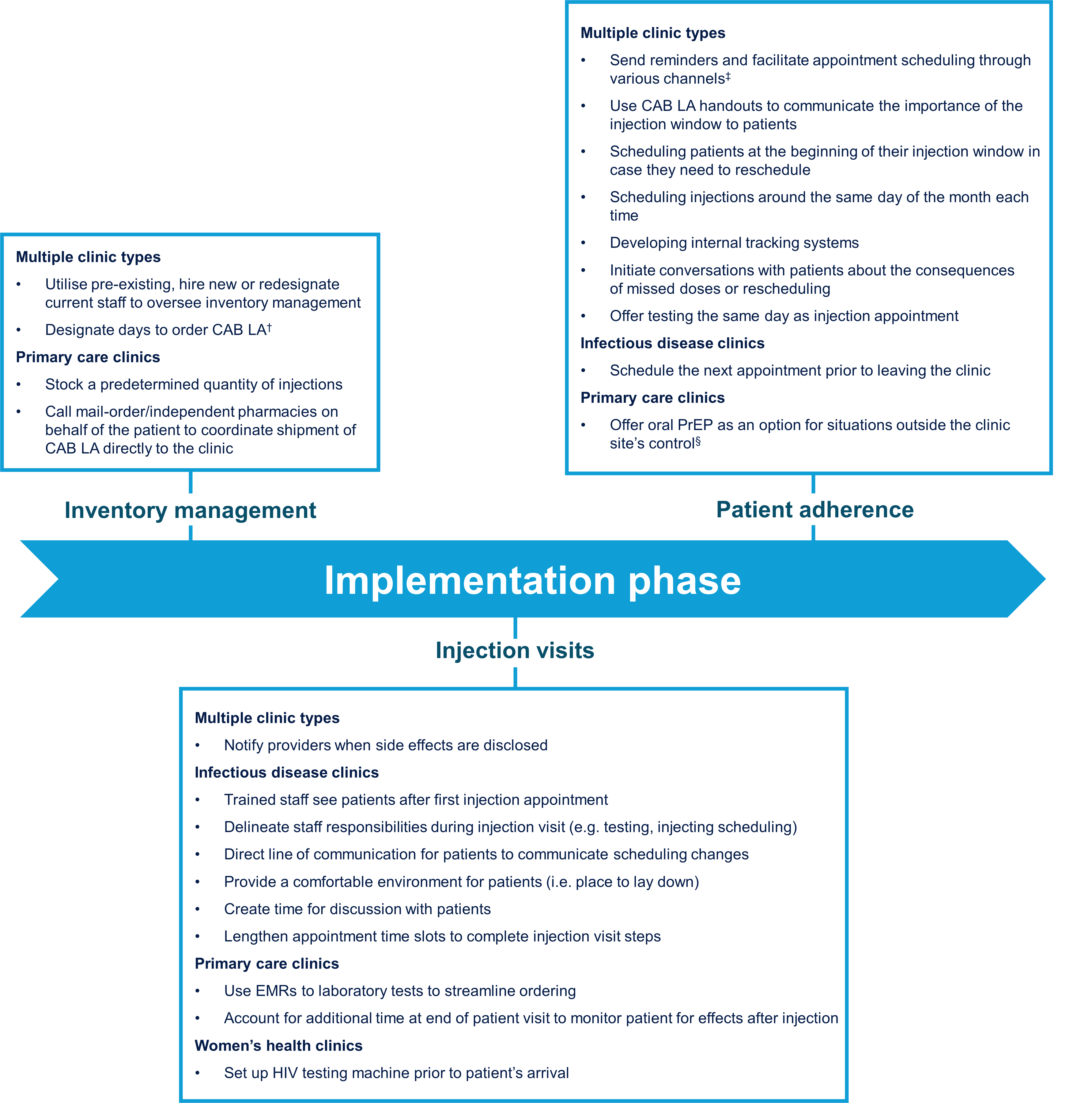
**

^†^I.e. first of the month for all patients with upcoming injections. ^‡^Text, email, etc.. ^§^E.g. changes in insurance.
CAB, cabotegravir; EMR, electronic medical record; LA, long-acting; PrEP, pre-exposure prophylaxis.

## Supplementary Table 1. Clinic-level characteristics

|  | **Clinics**  **(n=17)^†^** |
| --- | --- |
| Clinic demographics | |
| Estimated total number (median [IQR]) of Black  (cis- and transgender) women who: |  |
| came into the clinic/practice in the last 3 month for a clinical visit | 189 (87–790)^‡^ |
| received any type of PrEP — oral or injectable — at the clinic/practice in the last 3 months | 20 (5–40) |
| Estimated total number (median [IQR]) of Black transgender women who: |  |
| came into the clinic/practice in the last 3 month for a clinical visit | 9 (1–35) |
| received any type of PrEP — oral or injectable — at the clinic/practice in the last 3 months | 2 (0–6) |

^†^17 clinics provided quantitative data at Month 4 surveys for these specific questions. ^‡^Data from 16 clinics.
IQR, interquartile range; PrEP, pre-exposure prophylaxis.

## Supplementary Table 2. HCP demographics and characteristics (cross-sectional sample)

|  | **Cross-sectional sample**  **(n=99)** |
| --- | --- |
| Gender identity, n (%) |  |
| Cisgender male | 28 (28.3) |
| Cisgender female | 56 (56.6) |
| Transgender woman | 1 (1.0) |
| Nonbinary | 1 (1.0) |
| Other | 6 (6.1) |
| Prefer not to answer | 7 (7.1) |
| Age, median (interquartile range) | 43.0 (35.0, 52.0) |
| Race, n (%) |  |
| Asian | 3 (3.0) |
| Black | 45 (45.5) |
| Mixed race | 8 (8.1) |
| White | 31 (31.3) |
| Native American | 1 (1.0) |
| Other | 5 (5.1) |
| Prefer not to answer | 6 (6.1) |
| Role type, n (%) |  |
| Physician | 19 (19.2) |
| Advanced practice provider | 18 (18.2) |
| Medical assistant | 16 (16.2) |
| Administrator (office/clinic) | 8 (8.1) |
| Nurse | 11 (11.1) |
| Other role | 27 (27.2)† |
| Administers any type of injection, n (%) |  |
| Yes | 39 (39.4) |
| No | 60 (60.6) |
| Prescribes medication, n (%) |  |
| Yes | 37 (37.4) |
| No | 62 (62.6) |
| Medical specialty,^‡^ n (%) |  |
| HIV/infectious disease specialist | 24 (66.7) |
| Internal medicine/primary care/general doctor/family practitioner | 17 (47.2) |
| Women’s health/OBGYN | 3 (8.3) |
| Other^§^ | 2 (5.6) |

^†^Pharmacist (n=3), social worker/case manager (n=5), PrEP educator/navigator (n=4) and other (n=15).  ^‡^Responses are amongst the n=36 participants who responded “yes” to prescribing medications as part of their role. Participants could select ≥1 specialty.  ^§^Includes immunologists and other.
HCP, healthcare professional; OBGYN, obstetrics and gynaecology; PrEP, pre-exposure prophylaxis.

## Supplementary Table 3. HCP perceptions of implementation barriers before CAB LA implementation and 4 Months into CAB LA implementation (longitudinal sample)

| **Concern^†^** | **Baseline  (n=92)** | **Month 4  (n=92)** | **Absolute  % change** |
| --- | --- | --- | --- |
| *Pre-implementation phase* | | | |
| *Practice preparation* | | | |
| Staff time to provide counselling and support to patients, such as answering questions between visits and keeping patients motivated | 9.8% | 7.6% | –2.2% |
| Staff resourcing to ensure appropriate clinic flow | 8.7% | 6.5% | –2.2% |
| Ability to provide HIV screening as indicated at each injection visit and provide rapid intervention/follow-up based on results status | 3.3% | 3.3% | 0.0% |
| Staff preparation (i.e. education, training, in‑services) to implement CAB LA | 3.3% | 4.3% | +1.1% |
| *Patient identification* | | | |
| Patients’ willingness to travel every  2 months for an injection appointment | 26.1% | 14.1% | –12.0% |
| Patient’s lack of belief in efficacy of CAB LA | 14.1% | 6.5% | –7.6% |
| Identifying eligible individuals for CAB LA | 10.9% | 6.5% | –4.3% |
| Worried that a patient will feel stigmatised if offered CAB LA | 7.6% | 3.3% | –4.3% |
| *Benefits investigation and product acquisition* | | | |
| Cost of CAB LA | 43.5% | 34.8% | –8.7% |
| Cost of ancillary services to provide  CAB LA | 29.3% | 23.9% | –5.4% |
| *Implementation phase* | | | |
| *Injection visits* | | | |
| How to transition patients off CAB LA who want to stop taking it (e.g. should other PrEP be offered) | 10.9% | 6.5% | –4.3% |
| Management of scheduling injection appointments every 2 months during correct injection (i.e. dosing) windows | 8.7% | 3.3% | –5.4% |
| Difficulty of giving the gluteal  medial injection | 8.7% | 3.3% | –5.4% |
| Ability to manage patients attending CAB LA injection appointments with other care needs that need to be addressed | 7.6% | 5.4% | –2.2% |
| Managing the oral lead-in phase of CAB LA prior to starting injections  if needed | 4.3% | 3.3% | +1.1% |
| *Patient adherence* | | | |
| Risk of drug resistance for patients not adherent to injection visits | 40.2% | 26.1% | –14.1% |
| Patients’ ability to keep injection appointments | 38.0% | 25.0% | –13.0% |
| Keeping a patient engaged and motivated to continue CAB LA | 29.3% | 18.5% | –10.9% |
| Understanding how to manage patients who miss an injection dose | 10.9% | 4.3% | –6.5% |
| Ability to provide adherence counselling and support, when injection dosing visits are missed | 7.6% | 2.2% | –5.4% |

^†^Concerns were measured on a 5-point Likert scale (1 = extremely concerned, 5 = not at all concerned). Results presented here were rated by HCPs as “extremely concerned” or “moderately concerned.”
CAB, cabotegravir; HCP, healthcare provider; LA, long-acting; OLI, oral lead-in; PrEP, pre-exposure prophylaxis; STI, sexually transmitted infection.
